# Defining the water flow cues for navigation in migrating Atlantic salmon smolts

**DOI:** 10.1111/jfb.70004

**Published:** 2025-03-11

**Authors:** Mikolaj E. Kundegorski, Hannele M. Honkanen, Alastair Stephen, Colin J. Torney, Shaun Killen, Colin E. Adams

**Affiliations:** ^1^ Scottish Centre for Ecology and the Natural Environment, SBOHVM University of Glasgow Glasgow UK; ^2^ School of Mathematics and Statistics University of Glasgow Glasgow UK; ^3^ Scottish and Southern Energy Inveralmond House Perth UK

**Keywords:** anadromy, aquaculture effects, domestication, migration, salmon, water velocity

## Abstract

For migratory species, successful navigation is critical to fitness. In Atlantic salmon, for example, there is evidence that during migration from natal streams to the sea, passage through waters with poorly defined or mixed water velocity patterns may constrain directional navigation, causing individuals to become trapped or delayed in lakes or other bodies with slowly flowing water. In this study, we determined the minimum water velocities needed to elicit a behavioural response, in this case a change in the direction of holding position, in both wild origin and domesticated salmon smolts. Smolts required a directional flow in excess of 8.9 cm s^−1^ to exhibit effective directional orientation towards the current. Smolts of a domesticated farm origin exhibited a similar qualitative and quantitative response as wild fish. These results suggest that, in areas where the downstream migrating Atlantic salmon smolts pass through low‐directional water flow, it may be possible to manipulate directional flows above this minimum threshold, at least temporarily, as a management tool to increase migration success. This is likely to be particularly true where smolts are passing through dams, reservoirs or other impounded waters.

## 1 INTRODUCTION

Long‐distance migration is a common strategy found across a wide range of animal taxa, which often involves movement between breeding and feeding grounds (Dingle & Drake, 2007). Such migration is associated with a high level of risk, including predation by novel predators, exposure to disease and parasites and the chance that the migrant will not successfully reach the intended migration destination (Dingle, 2014). Overcoming this last risk is particularly challenging, especially for individuals undertaking the migration with no prior experience of the route (Adams et al., 2022). Therefore, it is clear that successful migration is dependent upon high‐quality navigation ability.

Animal navigation, especially in the case of long‐distance migration, is hugely complicated and still not fully understood (Alerstam, 2006; Mouritsen, 2018; Putman et al., 2014). What we do know suggests that migration is only infrequently a passive process, for example, where the animal relies on wind or water current to move. One of the few examples of passive migration among fishes appears to be the migration by the larvae (leptocephali) of the European eel (*Anguilla anguilla*) (Ginneken & Maes, 2005). However, even in marine environments, where there is scope for passive movement using currents and there is a specific end goal location, variation in current patterns means that a purely passive migration strategy is directionally unreliable (Putman et al., 2014). Active migration requires cues to identify the right time to start moving, an awareness of the surrounding environment, knowledge of when to stop moving and the ability to identify and interpret the correct directional cues for navigation (Åkesson et al., 2014). Some of the known mechanisms that animals use to orientate and navigate during migration include celestial, geomagnetic, visual and olfactory cues (Bolshakov et al., 2007; Cochran et al., 2004; Wikelski et al., 2015). The importance of these cues is well understood; however, the required intensity of the cues needed for successful navigation is largely unknown.

Migratory Salmonidae fish use several types of cues for navigation, including water flow, olfaction and geomagnetic maps (Madsen et al., 2019; Putman et al., 2014). During the early stages of marine migration, Atlantic salmon, *Salmo salar*, seemingly use surface currents for direction finding (Dadswell et al., 2010; Gilbey et al., 2021; Mork et al., 2012). Work on Chinook salmon, *Oncorhynchus tshawytscha*, and sockeye salmon, *Oncorhynchus nerka*, has shown that these fish also use a combination of magnetic intensity and inclination angle to navigate at sea and olfactory cues during their return journey to fresh water (Dittman et al., 1996; Drenner et al., 2018; Madsen et al., 2019; Putman et al., 2014). Rheotaxis, orientation to flow, is one of the main ways in which fishes respond to their environment. It is a flexible behaviour and used for many purposes, from maintaining position in flowing water to searching for food (Coombs et al., 2020). For juvenile Atlantic salmon, in common with many other salmonids, rheotaxis has a significant role in their feeding strategy during their first few years of life when it facilitates their drift‐feeding behaviour (Arnold et al., 1991; Holleman et al., 2022; Klemetsen et al., 2003). During the early stages of smolting in Atlantic salmon, this station‐holding behaviour alters and fish begin to lose positive rheotaxis to move downstream with the current. Initiation of the downstream migration by smolts is frequently associated with increased water flow, once a minimum temperature has been reached and day length increases (Connor et al., 2003; Hvidsten et al., 1995).

Evidence of the importance of water flow as a key navigational cue comes from investigations of Atlantic salmon smolt migration through standing waters (lakes and reservoirs). Multiple studies have shown that smolts experience very high migration failure rates and long passage times by successful migrants during lake migration compared with their passage in river channels (Aarestrup et al., 1999; Lilly et al., 2021; Schwinn et al., 2017, Schwinn et al. 2019). It has been suggested that this stems from the inability of smolts to find the outflow of lakes. This is evidenced by random and directionless swimming patterns undertaken by both smolts that migrate successfully and those that do not in these habitats (Hanssen et al., 2022; Honkanen et al., 2018; Honkanen et al., 2021; Lilly et al., 2021; Schwinn et al., 2017; Schwinn et al., 2018). One possible explanation for the apparently random pathway patterns is that high‐quality directional information, which the smolts require as a cue for navigation, is missing, or at least highly reduced, in lakes (Schwinn et al., 2017). Further evidence of the likely fitness cost of lakes comes from Hutchings et al. (2019) who found that of the 72 non‐anadromous salmon populations worldwide, 82% are found in catchments with lakes, suggesting that these habitats discourage migration.

Despite the well‐known importance of water flow as a directional cue during the riverine component of smolt migration, there is a paucity of information on the ability of salmonids to detect current and in particular the sensitivity of current detection that allows them to orientate. It has been suggested that juvenile salmonids can sense flow change that is as low as 0.4–1.0 cm s^−1^ (Gregory & Fields, 1962 in Enders et al., 2012), but there is very little information on the minimum water velocity that can be sensed by salmonids and which will initiate a response. In one of the very few studies on this topic Veselov et al. (1998) tested the minimum water velocity which ‘elicited movement of eyes, fins, or curving of the body, and resulted in the fish orienting into the current’ in Atlantic salmon. They tested alevin, fry, parr and smolt life stages by quickly accelerating the water velocity from 0 to 160–200 cm s^−1^ in a small chamber. They found that the minimum velocity that elicited such a response was ~4.3 cm s^−1^ for alevins (fish length 2.3–2.6 cm) but that this decreased to ~2 cm s^−1^ for fry and parr (fish length 4–8 cm) and then increased to ~5.5 cm s^−1^ for smolts (fish length 10–12 cm). Although detailed, this study did not address a common scenario where fish have to acclimatise in, and respond to, a heterogeneous flow environment.

Sensitivity and response to flow cues could be linked to their previous environment, so individuals that experience a range of naturally fluctuating flows may have a higher sensitivity threshold compared with farmed Atlantic salmon that are reared in controlled hatchery conditions with very weak flows. Farmed Atlantic salmon have been subjected to domestication selection for between 10 and 20 generations and are therefore genetically distinct from the wild populations (López et al., 2019). Many studies have shown differences between wild and farmed salmon in a range of characters such as growth rates, physiology, behaviour and gene transcription (Einum & Fleming, 1997; Fleming et al., 2002; Glover et al., 2017; Roberge et al., 2008).

Here we tested the minimum water flow rate that elicits a change in the direction of holding against the current in Atlantic salmon smolts in the seaward migration phase of their life cycle in a controlled environment experimental trial. A secondary aim was to test if Atlantic salmon of farm origin have retained the ability to use water flow as a cue for orientation and at the same level of sensitivity as their wild‐origin counterparts. Specifically, this study addressed three questions. (1) What is the behavioural response of salmon smolts to changing water velocity? (2) What is the minimum detectable threshold water velocity that elicits a change in behaviour? (3) Do wild and farmed smolts respond differently to changing water velocity?

## 2 METHODS

Behavioural trials were conducted at the Scottish Centre for Ecology and the Natural Environment (SCENE), University of Glasgow. The behavioural arena was part of an oval flume tank, 11 m long and 0.6 m wide. Water depth was ~20 cm, and a directional flow was created in the channel by water being drawn from a sump in the flume outside the experimental arena and pumped through directional nozzles back into the channel at the opposite end of the flume. Water in the flume tank was drawn from the nearby Loch Lomond and was maintained at ambient temperature (range: 6–13.6°C, water surface temperature determined using the dataset from Chin et al., 2017; these modelled data are in line with empirical data for the time of year these experiments were conducted) during the study. Behavioural trials took place in a straight section of the flume (1.6 m long and 0.96 m^2^), partitioned by screens through which water could easily flow. Trials were filmed from above using two time‐synchronised Raspberry Pi computers each connected to a Full HD near‐IR (day/night) camera with a recording frequency of 15 frames per second.

At the beginning of each trial, a single Atlantic salmon smolt was placed in the test area with a stable current (~20 cm s^−1^ in the middle of the channel), with flow in either the clockwise or counterclockwise direction for 20 min, to allow the fish to acclimatise to tank conditions and to orient to the flow. Typically, smolts would orientate head first into the water flow during this period. At the end of the acclimation period, the water flow direction was reversed for the remaining 5 min of the trial. To achieve this, the direction of flow from the pumps creating the directional flow in the test arena was reversed at a single point in time. The effect in the arena was that water speed decreased slowly and then reversed direction, slowly increasing in the experimental area to a velocity that was close to the original velocity but in a direction ~180° from the original current direction. Due to the size of the flume and the volume of water it contained and despite that flow velocity delivered by the pump was at a maximum from the moment of flow reversal, it took up to ~3 min after directional change for the flow velocity to return to its original velocity (but in the opposite direction) at the location where the fish was holding. For each test fish the lowest water velocity that initiated a behavioural flow reorientation response in the test fish was determined (*V*_min_). This was defined as the water velocity at which the smolt changed orientation (by turning 180°) to face the new current direction. To measure this, the precise time at which the smolt reorientated (the reaction time) and the exact location within the trial arena where this occurred (determined from scaled coordinates taken from video footage) were recorded. After the trial with a fish, a velocimeter (Hontzsch Flowtherm NT) was placed at the position at which the smolt in that trial was located. The change in water direction was then replicated without the presence of a fish. *V*_min_ was measured as the mean water velocity measured over a 10‐s period (5 s before and 5 s after the time of the initiation of the reorientation by the test fish). The average response time for a fish was 1.5 min, and for most of the measured points, flow stabilised in the new (opposite) direction after ~3 min. In addition to this, the velocity of the stable current before and after flow direction change was also measured at the position where the fish was holding. This varied around a mean of 12.9 cm s^−1^ (SD = 5.2, range: 2.0–22.4 cm s^−1^). All velocity measurements were made at ~2 cm from the bottom of the flume tank to correspond with the depth at which fish would be experiencing flow when holding at the bottom of the tank. Across all fish, in all trials, the mean velocity to which test fish were exposed during the acclimation period and after flow reversal was 12.6 cm s^−1^ (SD = 5.3).

Three groups of Atlantic salmon smolts were used in trials conducted in three separate periods; two fish groups were of fish farm origin, and the third comprised wild‐caught fish. In the first set of trials (August 2019), farmed fish originating from an indoor, tank aquaculture facility (supplied by MOWI's, Lochailort Hatchery) were tested. These fish (*N* = 52 randomly selected individuals, from a larger group with a mean weight of 120 g [SD = 22] and a fork length of 20.7 cm [SD = 1.3]) were transported to SCENE as parr and allowed to develop to the smolt stage (assessed using the criteria of Gorbman et al., 1982) before trials were conducted (water temperature during these trials was 13.6–16.1°C). The second set of farmed fish trials were conducted in March 2021 (water temperature during these trials was 6.0–6.7°C), using fish originating from an outdoor freshwater lake cage farm system (supplied by MOWI: Loch Arkaig). These fish (*N* = 58, mean weight of 44 g [SD = 14], mean fork length of 16 cm [SD = 1.5]) had already reached the smolt stage or were very close to smolting on collection. Trials of farmed fish commenced once they began smolting and were acclimatised to a spring‐like photoperiod (16:8), mimicking that of their natural migration period. We also verified the initiation of migration by observing a consistent downstream movement for fish in holding tanks from those groups before our trials. Initially, both farmed fish groups were raised under constant light to rapidly increase their growth, a practice diverging significantly from their wild counterparts in terms of seasonal and temperature conditions.

The third set of trials were conducted in May 2021 on wild salmon smolts collected from the River Gryffe, Scotland (55°51.9′ N, 004°31.1′ W). Smolts were captured in a rotary screw trap during the smolt run (*N* = 31 individuals with a mean weight of 24 g [SD = 6] and a mean fork length of 13 cm [SD = 1]). These fish were transported to SCENE in oxygenated bags (transport time ~1 h) and then placed in holding tanks in which they were held for at least 2 h before trials. The daily temperature during those trials ranged 7.7–8.1°C.

To account for any potential bias in directionality, the initial flow direction to which fish were exposed during the trials was alternated from clockwise to counterclockwise.

The flow conditions experienced by fish during holding in the experimental area varied between experiments. Water turbidity varied with the conditions in the water source, and for technical reasons the pump creating the flow was changed between experiments (although with the same nominal output).

Despite this variation, fish exhibited positive rheotaxis when holding position in the behavioural arena. A flow profile of the flume section where experiments were conducted showed a high coefficient of variation of 0.42 (mean = 18.25, SD = 7.66). All of the wild fish experienced the same flow conditions except for the initial flow direction. The flow value experienced at the point where fish were holding prior to current switching was tested as a possible explanatory variable influencing the current at which a behavioural response occurred.

During the trials, four behaviours were categorised during the 5‐min period when the flow direction was changing (see Table 1). For two of these, ‘swim off and constant movement’ were behaviours that were uninformative in respect of the main aim of this study, which was to determine a response to flow direction and velocity experienced. This is because both of these behaviours could be responses to other environmental or internal cues. Thus, for example, the behaviour swim off could be a response related to the investigation of a possible food item in the water column. The two behavioural categories that were informative in relation to flow cues and were thus analysed here were change of direction and no reaction. We classified cases where the fish was in movement throughout the experiment (constant movement) or initiated movement before any other response to flow direction was classified (swim off) as ‘other behaviours’. These cases were not statistically analysed, but numbers are provided for insight into the ranges of behaviours observed.

**TABLE 1:** Classification of behaviour of the fish during the 5 min from initiation of change in water flow direction.

Response to flow	Number of samples	Name	Description
Yes	62	Change of direction	A change in fish orientation indicated by an ~180° switch in their holding position to a positive rheotaxis after directional change in flow conditions.
No	24	No reaction	The fish did not seem to respond to flow direction change behaviourally either with a change in orientation direction or by moving position.
Other behaviours	24	Swim off	The fish although holding station at the start of the trial initiated movement away from its holding position without directional flow change.
	24	Constant movement	The fish kept moving position throughout the trial showing no clear response, which could be related to the directional current change.

*Note*: During this period, water velocity initially decreased and then reversed direction as a result of the experimental manipulations. Only one of these behaviours, change of direction, was a clear response to the cues provided by flow direction change. Constant movement and swim off comprised responses to unknown cues and thus were combined as other behaviours.

We analysed the data using R (R Core Team, 2021) using generalised linear models (GLM). Seven trials were excluded where experimental measurement error occurred. A behavioural reaction to flow change in the trial was modelled as a binomial model with change of direction or no reaction as the two outcomes. Fish group (indoor tank farmed, outdoor cage farmed and wild) and general flow conditions (direction and high/low flow strength) were considered as variables together with their interactions. Trials where the fish exhibited constant movement and swim off behavioural categories were excluded from this analysis.

The minimum flow velocity (*V*_min_) which resulted in a behavioural response was modelled for individuals, which successfully changed their direction of holding using a γ distribution with a logarithmic link function. To compare nested models we primarily used ANOVA with likelihood ratio test. Bayesian information criterion (BIC) and AIC were used to quantify the quality of a model fit with likelihood while penalising model complexity to compensate for overfitting. We report both values to compare the quality of fit for all models. For post hoc analysis we used Tukey's range tests. We included fish group (smolt origin), the magnitude of stable flow at the fish location prior to change in orientation direction and their interactions as explanatory variables.

## 3 RESULTS

**FIGURE 1:**
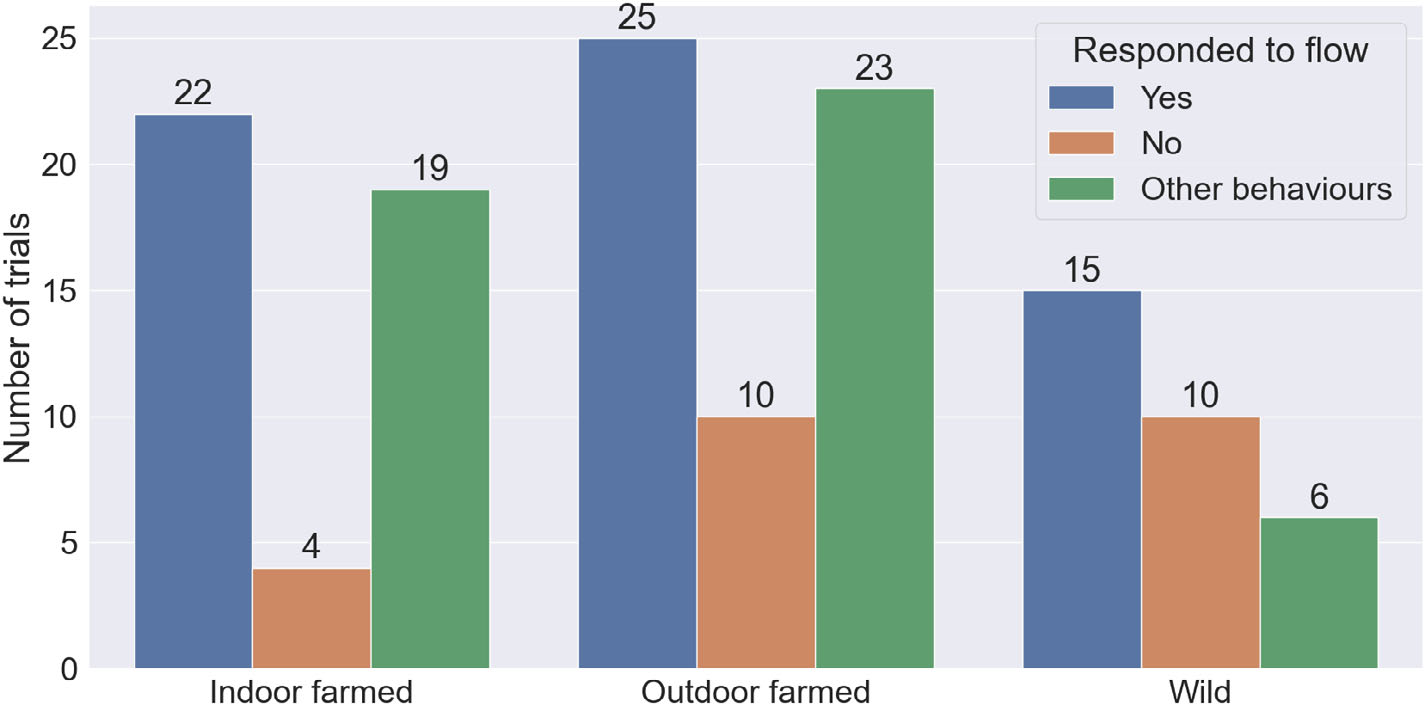
The number of trials in which fish from different origins (indoor [tank] farmed, outdoor [cage] farmed and wild) responded to the reversal of flow in the experimental area by switching direction. A clear response to flow was the most common outcome in all groups.

**FIGURE 2:**
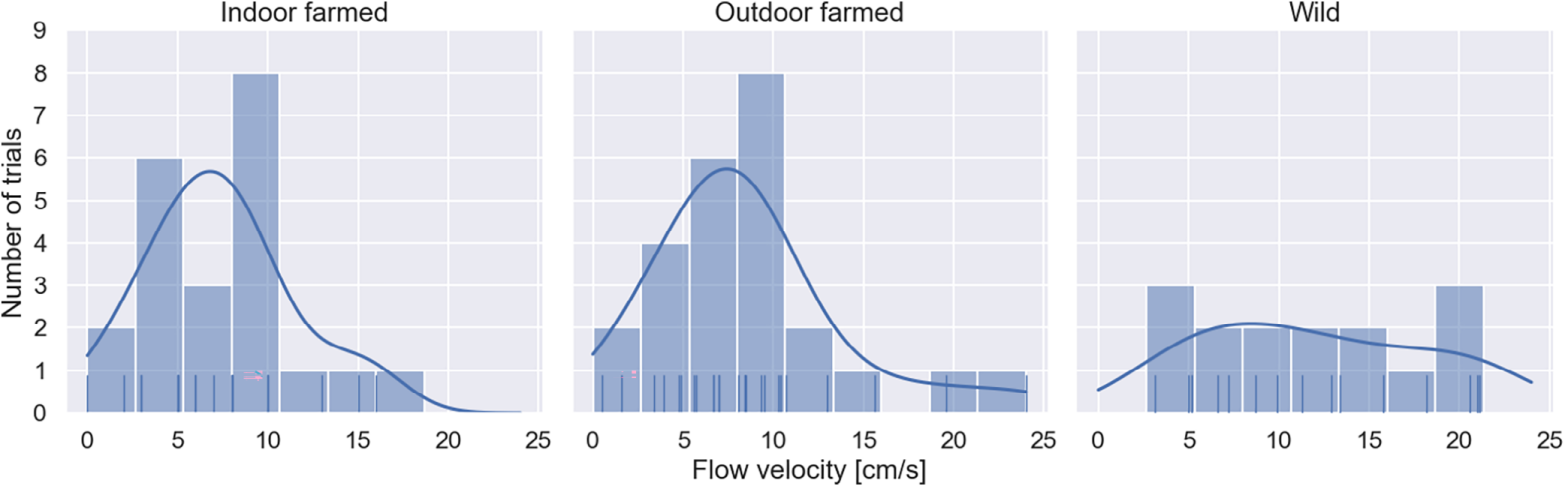
Histogram and kernel density estimate (KDE) plot exhibiting the minimum flow velocities (*V*_min_ [cm s^−1^]) that prompted a change in directional orientation across three fish groups. The vertical ticks along the *x*‐axis represent individual observations contributing to the histogram formation. Each panel corresponds to one of the fish origin groups: indoor (tank) farmed, outdoor (cage) farmed and wild.

In no trial did fish exhibit negative rheotaxis before flow reversal. Changing directional water flow caused change of direction or no reaction in 86 fish (64% of all fish successfully tested), whereas for 48 fish (36%) other behaviours were recorded (Figure 1). In these cases fish either were in movement during the flow reversal (constant movement) or did not change orientation when initiating a movement (swim off; see Table 1). Change of direction was the most common response to flow direction change across all three fish groups, exhibited by 72% of the fish where behaviour was classified (62 individuals) from across all experiments (Figure 1). The proportion of trials where the response was other behaviours was lowest in wild smolts, whereas it was similar in both groups of farmed fish (Figure 2). The binomial model with change of direction/no reaction as the response variable, with and without fish group as an explanatory factor, showed that there was no significant effect of fish group (ANOVA: *χ* ^2^ = 3.98, *df* = 2, *p* = 0.137, models fit: BIC = 111 and AIC = 104 with fish group variable, and BIC =106, AIC = 104 without).

Using a γ‐distributed GLM we estimate that the mean minimum threshold flow (*V*_min_) for directional change across all three fish groups was 8.9 cm s^−1^ (95% CI: 7.7,10.4) (Figure 2). The mean *V*_min_ for the two groups of farmed fish were 8.5 (95% CI: 6.7,10.9) cm s^−1^ for the outdoor cage farm–origin smolts and 7.3 (95% CI: 5.8,9.2) cm s^−1^ for the indoor tank farm–origin fish. The mean *V*_min_ for the wild fish was slightly higher at 12.0 (95% CI: 9.2,15.6) cm s^−1^. The full model, including the flow rate experienced by fish during the initial holding period and its interactions with fish group (AIC = 401, BIC = 416), exhibited no effect of fish group on the minimum flow velocity required to initiate a reorientation towards the new current direction. However, an ANOVA analysis with likelihood ratio test implied that fish group was a significant covariate (*χ* ^2^ = 2.231, *df* = 2, *p* = 0.031), whereas flow velocity at the point of holding and its interaction with fish group were not significant (χ^2^ = 0.096, *df* = 1, *p* = 0.59, and *χ* ^2^ = 0.451, *df* = 2, *p* = 0.51, respectively).

The simpler model with fish group as the only dependent variable shows a better balance between the fit and the number of explanatory variables (AIC = 396, BIC = 405), and a post hoc statistical analysis with Tukey's range test showed that the only statistical difference in flow rate required to initiate a change in orientation was between the wild fish group and the indoor tank–farmed fish (ratio = 0.61, *p* = 0.031). As some variables such as water temperature, time of the year, light and type of pumps used are highly correlated with the group of fish and thus cannot be modelled directly, we cannot rule out that fish group is only a proxy for some other variable.

To examine more closely the effect of fish group (the origin of the experimental animals) while minimising the effect of these potentially confounding variables, we analysed a subset of 15 fish drawn from the group originating from an indoor cage farm and all 15 wild smolts that responded to flow change and were closest in their conditions (identical pump outputs, exposed to temperatures within a range of 2.1°C, no light manipulation). In this subset we found that neither previous flow conditions (*χ* ^2^ = 0.432, *df* = 1, *p* = 0.23) nor group of fish (farmed or wild) (*χ* ^2^ = 0.202, *df* = 1, *p* = 0.41) had a significant effect on the *V*_min_, the water velocity at which fish reorientated.

According to the BIC, the intercept‐only model (AIC = 396, BIC = 400) provides the best balance between fit and complexity, highlighting a lack of strong evidence for differences among the groups studied.

## 4 DISCUSSION

Here we show that the water velocity required to initiate a behavioural response resulting in a direction of orientation change in Atlantic salmon smolts is relatively low, with a mean *V*_min_ of 8.9 cm s^−1^ (~0.6 body length s^−1^) across all tested groups. Wild fish and the two groups of farmed Atlantic salmon smolts behaved similarly, although the wild fish had a slightly higher (but non‐significant) mean *V*_min_ value than the farmed fish, suggesting that the farmed fish, especially those from indoor tank facilities, might be marginally more sensitive to the velocity change. The wild fish group exhibited the least pronounced peak in the *V*_min_, which elicited a behavioural reorientation response, and there was considerable variation around the mean response for all groups (Figure 2). It is self‐evident that the fish in this study must be able to detect a change in current direction at a velocity lower than that at which they respond to that current. It is very possible that there is between‐individual variation in the time between a directional change in current and a behavioural reorientation and that this may in part be responsible for the observed variation in *V*_min_. Despite this, the difference between the determined mean *V*_min_ and the upper end of the range of *V*_min_ detected by a behavioural reorientation response is only a twofold difference.

It is frequently reported that migration is associated with the highest mortality rates during an animal's life cycle (Cresswell et al., 2011; Guillemain et al., 2010; Owen & Black, 1991; Sillett & Holmes, 2002; Strandberg et al., 2009). This is also true for salmonids; the downstream migration of Atlantic salmon smolts is associated with variable but relatively low survival, with mortality rates of between 0.3 and 7.0% km^−1^ reported during downriver migration and between 0.6 and 36% km^−1^ during the early phases of marine migration (Thorstad et al., 2012). Especially high mortality has been observed at the confluence of river and lake, likely due to predation, highlighting the necessity for reducing time spent finding the route out of the man‐made reservoirs (Kennedy et al., 2018). During this initial and risky part of the Atlantic salmon migration, the evidence suggests that the most important cue for movement and migration is water flow (Otero et al., 2014; Scruton et al., 2003). Therefore, the fish's ability to detect this cue and also to react to it correctly is vitally important for successful migration. In rivers with clear and reliable water flow cues, the migration is consistently unidirectional (Thorstad et al., 2012). However, water flow–related directional cues become unreliable and poorly defined when downstream passage is through habitats that do not have strong, unidirectional currents, such as lakes, reservoirs and riverine areas near barriers (Honkanen et al., 2021). Multiple studies have shown that the lower water discharge, especially near barriers, contributes to delays and failure of migration (Gauld et al., 2013; Serrano et al., 2009). In this study, Atlantic salmon smolts were clearly responding to changes in current velocities, although there was interindividual variation (range: 0–24 cm s^−1^). The wild fish in this study were from a population that does not have any standing waters or in‐stream barriers on their migration route. It would be an interesting comparison to study populations from rivers that do and do not flow through a lake.

There is only one similar study that has investigated the flow sensitivity of juvenile Atlantic salmon in an experimental setting. Veselov et al. (1998) reported *V*_min_ values of ~5.5 cm s^−1^ for Atlantic salmon smolts. Their results show a broadly similar but a slightly lower threshold for detection than we found in the study presented here. This could, however, be due to a difference in the behavioural cue that was used to determine a flow change response in the experimental fish. In the Veselov study it is slightly unclear whether they measured the directional flow at the first signs of a behavioural response or when the orientation change actually took place. They also found that the minimum velocity threshold for a response (*V*_min_) value was lower for fry and parr than for alevin and smolts, suggesting that *V*_min_ is not simply related to fish size and may be connected with developmental stage, possibly reflecting the development of the sensory organs and behavioural characteristics at different life stages in this species. They also found seasonal variation in the responses, with *V*_min_ much higher at colder temperatures (Veselov et al., 1998).

Another interesting finding from our study is that the wild and farmed fish (from indoor [tank] and outdoor [cage] farm facilities) responded to changing current similarly. Thus, despite four or more decades of selection under domestication, farmed fish smolts have not lost the ability to respond to directional orientation at low flows that is found in wild fish. This is somewhat surprising as several studies have highlighted differences in behaviour and physiology between wild and farmed Atlantic salmon (Fleming et al., 1996; Houde et al., 2010; Jutila et al., 2003). When testing a sensory response to flow, it might be reasonable to postulate that farmed fish might have a higher *V*_min_ threshold, considering that they have been reared in an environment with mostly uniform flows and arguably no fitness benefits (i.e., higher food intake, dominance) for higher flow sensitivity. Alternatively, if farmed fish are exposed to low water velocities (which might well be the case in fish from outdoor cages in a lake), they may be more sensitive to changes than wild fish that experience considerable variation in flow conditions in their natal rivers.

There is clear management interest in the minimum water velocity cues that are required for fish navigation and therefore successful migration. Many migratory fish use rheotaxis as one of their main navigational cues during their early migration, and therefore water velocities that are of sufficient magnitude and directionally stabile are required for successful migration. However, it is likely that man‐made reservoirs have an added layer of complexity associated with migration, with altered hydrology near the impounding dam and the challenge of identifying any natural or man‐made fish passage into the efferent river at or near the dam (Babin et al., 2020; Havn et al., 2018). Difficulty finding an efferent passage into the river downstream can lead directly to mortality, or it may result in an additional cost incurred through a delay in migration, such as mortality via predation or smolts missing the physiologically suitable time period for entering the ocean (McCormick et al., 1998). However, where water velocities can be manipulated in standing waters, for example, where there are hydropower facilities with scope for at least partially influencing the hydrological conditions within a reservoir, it may be possible to create conditions in reservoirs that facilitate successful smolt migration. This approach was demonstrated by Xu et al. (2017) who modelled flow velocities across a large reservoir in China. Using 20 cm s^−1^ as the minimum flow requirement required for migratory fish to detect and follow a water velocity cue, they found that although most of the route through the reservoir (68%) met the minimal detection limit, there were two sections that fell below this threshold and thus constituted an invisible migration obstacle for fish. This sort of hydrological modelling in conjunction with the water velocity detection thresholds identified in our study has considerable potential for practical management. For example, such a detection threshold may aid in predicting where and when migrating salmon smolts may be making navigation decisions based on water flow and thus has the potential for manipulation in managed hydropower schemes.

## AUTHOR CONTRIBUTIONS

Concept: C.E.A., M.E.K., C.J.T., S.K. Data collection: M.E.K. Data analysis: M.E.K. Writing – original draft: H.M.H. Writing – review and editing: M.E.K, C.E.A, S.K., C.J.T., A.S. Funding acquisition: A.S., C.J.T.

## Supporting information


**Data S1.** kundegorski_figure 1.

**Data S2.** kundegorski_figure 2.
